# Genotypic Analysis of *E. coli* Strains Isolated from Patients with Cystitis and Pyelonephritis

**Published:** 2012-07-30

**Authors:** M Anvarinejad, Sh Farshad, R Ranjbar, G M Giammanco, A Alborzi, A Japoni

**Affiliations:** 1 Prof. Alborzi Clinical Microbiology Research Center, Shiraz University of Medical Sciences, Shiraz, Iran; 2 Molecular Biology Research Center, Baqiyatallah University of Medical Sciences, Tehran, Iran; 3 Department of Health Promotion Sciences, University of Palermo, Palermo, Italy

**Keywords:** Uropathogenic Escherichia coli, Pyelonephritis, Cystitis, Electrophoresis, Genetic patterns

## Abstract

**Background:**

Urinary tract infection is the most common health problem affecting millions of people each year, mainly caused by a large genetically heterogeneous group of Escherichia coli called uropathogenic *E. coli* This study investigates the genotypic analysis of *E. coli* strains isolated from patients with cystitis and pyelonephritis.

**Methods:**

During 2008-2009, 90 *E. coli* strains were analyzed, consisting of 48 isolates causing pyelonephritis in children and 42 isolates causing cystitis. Having identified the strains by standard methods, they were subtyped by pulsed field gel electrophoresis (PFGE) and their corresponding patterns were compared using dendrogram.

**Results:**

Sixty five PFGE profiles were obtained from the genome of *E. coli* strains by this genotyping method. Thirty six and thirty three patterns were obtained for pyelonephritis and cystitis, respectively. Most strains exhi-bited twelve and thirteen bands and the patterns with eight or nineteen bands had the lowest rate. Genome sizes of the strains were between 1610-4170 kbp.

**Conclusion:**

With due attention to these results, genetic patterns showed that the strains had different clonalities and it could be suggested in some cases that the strains causing pyelonephritis or cystitis have common patterns and different diseases could be explained by different gene factors.

## Introduction

Urinary tract infections (UTI) including pyelonephritis and cystitis are the most common extra-intestinal infections. Uropathogenic Escherichia coli (UPEC) is the most frequent agent causing UTI in adults and children.[1][2][3] Accumulation of bacteria occurs in different parts of the urinary tract tissues. UTI typing is based on the location of the tissue; if the colonization occurs in the lower urinary tract, it is called cystitis that happens in all ages and especially in females, compared to males. Symptoms of cystitis include pain above the pubic, urinary urgency and dysuria. If the bacteria involve the upper part of urinary tract, it is called pyelonephritis or kidney infection, which is accompanied by fever, nausea, and vomiting and flank pain.[4][5] Prevalence rate of the UTI usually depends on sex and age. In the neonatal period and first year of life, boys are more predisposed to these infections, due to birth defects and reflux vesicourethral. After 12 months UTI infections are limited to the girls.[5][6]

In the emergence of associated clinical syndromes, host and organism related factors could play an important role.[3][7] Different virulence factors have been involved in the colonization stage and defeating the host defensive system which results in the invasion of urinary tract.[8][9] Yet, none of these factors or any combination of them is known to be able to be involved in various clinical syndromes. Also, it is not clear whether all UPEC strains are capable of causing both cystitis and pyelonephritis or certain strains are associated with certain syndromes.[4][7] In the last decade especially during 2003 to 2007, many scientists were trying to identify genome and virulence factors of *E. coli* Considering these studies and according to the genetic diversity of the organism, no new virulence gene has been discovered yet and the pathogenesis of UTI remains unclear.[10][11][12] So, the results of molecular studies can be helpful in selection of the strains and the dominant gene locations. In other words, among the existing genotyping methods in molecular epidemiological studies of bacterial isolates, pulsed field gel electrophoresis (PFGE) is superior and gold standard because of its high discriminatory power of the isolates.[13][14][15][16][17] Some researchers have used this technique to evaluate the patterns of UPEC. Ejrnaes et al. demonstrated 15-20 distinct bands with fragments of 50-1200 kbp in molecular typing of UPEC strains.[18] The aim of the present study was to determine the genetic diversity of the *E. coli* isolated from children with urinary tract infections including cystitis and pyelonephritis using PFGE, a method widely used for its progress in standardization of electrophoresis conditions. In addition, to provide a reliable method in differentiating the isolated strains and whether all UPEC strains are capable of causing both pyelonephritis and cystitis, PFGE patterns were used in this study.

## Materials and Methods

*E. coli* strains were isolated from urine samples of children who presented at Motahary Hospital, Jahrom, Iran. UTI diagnosis was established by the hospital physicians based on the clinical symptoms and laboratory findings. *E. coli* isolates were identified by standard methods.[19] As the cases considered in this study were only the patients with community acquired UTI, the exclusion criteria were recent antibiotic use during 28 days ago and nosocomial infections which were defined as infections that were noted 48 h after admission or within 4 weeks after a previous discharge.[20] Positive urine cultures were defined by a bacterial growth >10^5^ colony forming unit/ml.

This procedure was designed based on previously reported protocol by Ejrnaes et al. with some modifications.18 Briefly, the isolates were grown overnight on blood agar plates at 37°C. In order to protect the DNA against breakage and to allow the free flow of lytic solutions, the bacteria were incorporated into agarose plugs, as described below. About three loops of bacteria were washed in 1 milliliter saline to obtain an optical density of 0.7 at wavelength of 610 nm and resuspended in 1 milliliter TE buffer [10 mM Tris HCl (pH=8.00), 100 mM EDTA] and incubated at 50°C in a water bath for maximum 15 minutes. Chromosomal DNA was prepared in solid agarose plugs by mixing 1 ml of bacterial cell suspension with an equal volume of 2% low melting agarose (Fermentase, Lithuania). Following the overnight incubation at 54°C in lysis buffer [50 mM Tris HCl (pH=8.00), 50 mM EDTA, 1% laurylsarcosine, 1mg/ml of proteinase K], the DNA plugs were washed four times in TE buffer for 30 minutes at 50°C and three times in distilled water. One third of each plug was cut and transferred to a tube containing XbaI restriction enzyme (Fermentase, Lithuania) according to the manufacture's instruction and remained overnight at 37°C.

DNA preparations were put in the wells of an agarose NA (molecular grade, Amersham Bioscience, Sweden), and covered with 0.5X TBE buffer (Trise base, Boric acid, EDTA, pH=8.3) and then were run in a homogenous electric field (Amersham Bioscience, Sweden). The electrophoretic conditions used were as follows; initial switch time: 5 seconds, second switch time: 20 seconds, final switch time: 40 seconds, temperature: 12°, run time: 33 hours, angle: 120°, gradient 6 v/cm. In each set, 1000 bp lambda ladder (Biolabs, New England) was used as DNA marker. After electrophoresis, the gel was stained in ethidium bromide and then photographed.

Photocapt software (Version 10.01, Vilber-Loumart, France) was used to determine the molecular weights of the sample profiles. The sizes of DNA fragments were determined according to the DNA marker. In each profile, the bands were recorded as number 1 for present or zero for absent. Consequently, the data set was used to calculate pair-wise similarity coefficient following the Jaccard method. To generate a dendrogram using average linkage procedure, the analysis of the similarity coefficients matrices were performed using unweighted pair-group method analysis (UPGMA).[21] To calculate correlations among the variables, the standardized data matrices were used. These correlations were subjected to Eigen Vector analysis to evince the first three uttermost elucidative principal components. To study the patterns of variations which were observed among the isolates, the three principal components were plotted. The NTSYSpc software (Version 2.02i, Exeter software, NY, USA) was used to conduct all the numerical analyses.

## Results

Ninty strains of *E. coli* were isolated from children with UTI, aged 1 month to 14 years ( mean 21.8±26.9 months), 60 females (62.5%) and 36 males (37.5%). Among the patients, 42 (46.6%) of cases had cystitis and 48 (53.3%) of the cases were diagnosed as acute pyelonephritis. Pyelonephritis was more prevalent in girls in comparison with boys (63.2% vs. 36.4%, p=0.04).

**Fig. 1:**
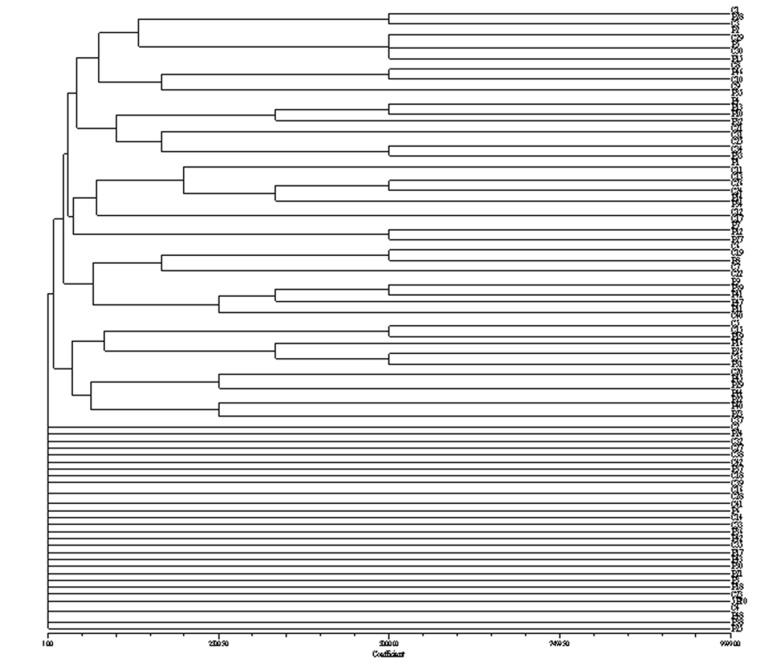
A dendrogram showing relationships among *E. coli* isolates from children with pyelonephritis (P) and cystitis (C).

Genomic DNAs of 90 strains of UPEC were restricted by XabI and analyzed by PFGE. All the strains were typable and reproducible banding patterns were obtained when analysis was repeated twice. Totally, sixty five PFGE profiles were obtained from the genome of UPEC strains based on drawn dendrogram (Figure 1). They were named from S1 to S65. Patterns including the most abundant band patterns were 13 and 12 in the 25.5% and 23.3% of isolates, respectively and the patterns with 8 or 19 bands had the least percentage. Pattern P2 was the most repeated pattern throughout the samples (n=5, 5.5%). The size range of the bands was from 2 to 660 kbp. The 520 kbp fragment consistently was present in all the pyelonephritic isolates, but not seen in the strains causing cystitis. On the contrary, the 310 kbp fragment was detected in all of the cystitis causing strains, but not found in the pyelonephritic strains. The genome size of the strains causing cystitis and pyelonephritis were 1630-3740 kbp and for isolates were between 1610 and 4170 kbp, respectively.

**Fig. 2:**
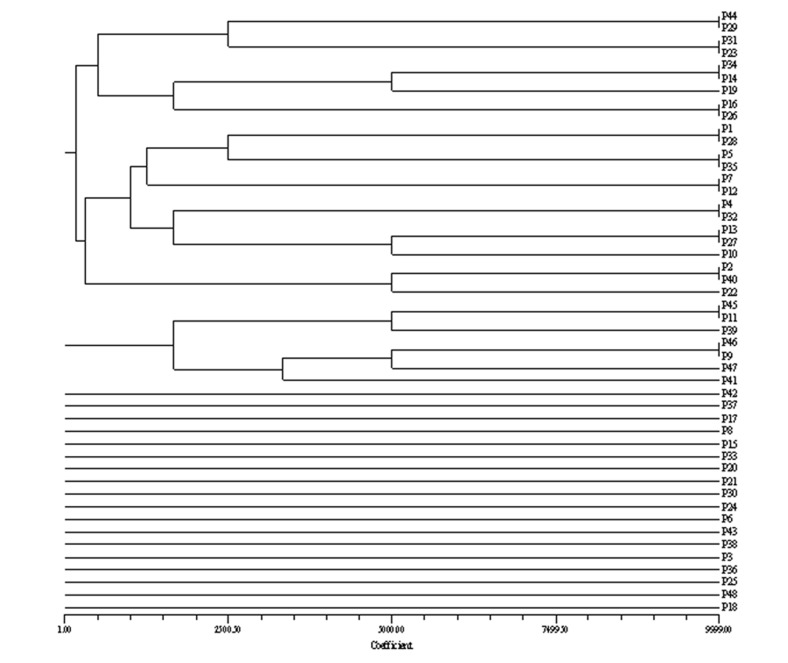
A dendrogram showing relationships among *E. coli* isolates from children with pyelonephritis (P).

**Fig. 3:**
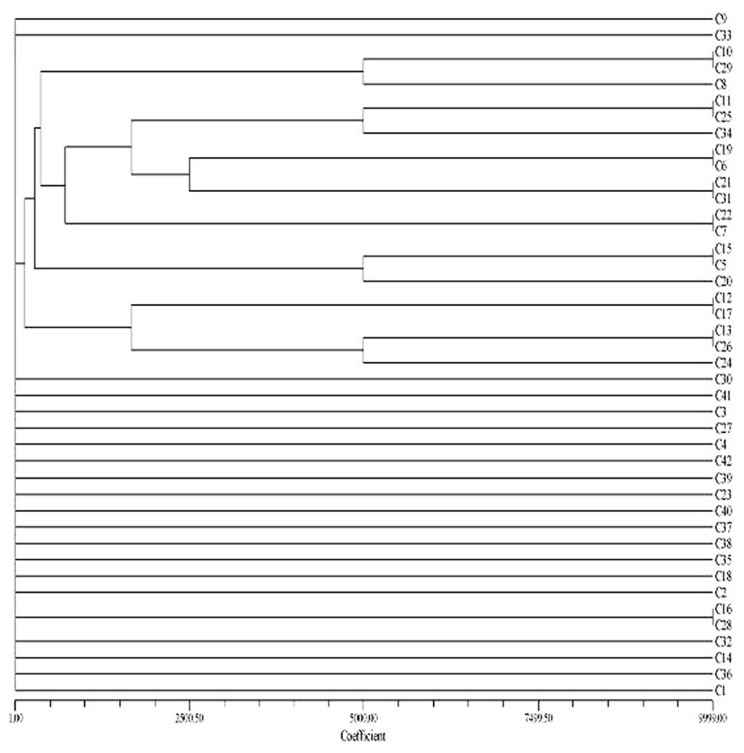
A dendrogram showing relationships among *E. coli* isolates from children with cystitis (C).

Thirty six PFGE profiles were obtained from the genome of *E. coli* strains causing pyelonephritis based on drawn dendrogram (Figure 2). Among 42 isolates of UPEC causing cystitis, 33 genetic patterns were detected (Figure 3) and the number of bands ranged from 8 to 19 for the isolates causing pyelonephritis and 9 to 16 for those causing cystitis.

## Discussion

*E. coli* is a microbial intestinal flora whose diversity cause large uncertainties in the identification of new genomes.[7][11] It is not clear whether each syndrome of urinary tract infection is caused by certain isolates or similar strains originating from the same clonality.[4] Most strains of *E. coli* are not threats as long as living in the gut, but if they enter the other parts of body such as genital tract, appendix, gallbladder, eyes, respiratory tract and conjunctiva, they may cause bacterial infection.[22] UPEC could involve the upper and lower urinary tract infections in children and differentiating infections in these sites by systemic and clinical signs or laboratory findings is not always helpful.[6][23][24] Therefore, to provide a reliable method of distinguish the isolates and discovering dominant gene locations and whether all UPEC strains can cause pyelonephritis and cystitis, the genetic patterns of strains were evaluated in this study.

Since the genetic characteristics show fewer changes,[25][26] they can be used to check the genetic pattern. Among the genotyping methods, PFGE is the most common technique used to study the relationship between strains in different regions.[27][28] In fact, comparing pulsed field gel electrophoresis pattern is a useful method for obtaining the possible association between the isolates of a species.[29] The development of PFGE typing methods based on fingerprinting of bacterial genome has given valuable tools to confirm the relationship among outbreak strains.[27] In the current study, we applied PFGE followed by XbaI restriction digestion of chromosomal DNA to determine the genetic relatedness among UPEC isolates. This enzyme is the most frequently used restriction enzyme for UPEC outbreaks,[30][31] and was successfully applied here to discriminate between the isolates from the infected children in Jahrom, southern Iran and generated different bands with diverse molecular weights. Number of DNA bands were produced in this study by using the prenominate enzyme; for pyelonephritis strains were between 8 and 19 bands and for strains causing cystitis were between 9 to 16 bands. Molecular weight bands were also very different for each syndromes; 2 kbp to 660 kbp. Since the samples were collected in a year and from different parts of population, these results were predictable. Other studies have shown different bands with high diversity of molecular weights. Kawamori et al. reported 19-24 DNA bands with molecular sizes of 30 to 500 kb, in molecular typing of Japanese *E. coli* isolates.[32] Ejrnaes et al. reported 15-20 distinct bands with 50-1200 kb molecular weights by PFGE typing of UPEC strains.[18] In Northern Ireland, Watabe et al. in an epidemiologic study of *E. coli* isolates, observed 15 DNA fragments using PFGE.[29]

One of the other main PFGE applications is the estimation of bacterial genome size determined by adding the respective generated bands. By obtaining the different genome sizes of strains, the genetic diversity of the species can be traced. Previous studies using either PFGE or whole genome sequencing also have revealed a diversity of bacterial genome sizes, ranging from as low as 0.5 Mbp to as high as 10 Mbp.[33] In the present study, such sizes of the genome were widely different, that is, for the strains causing pyelonephritis, they were between 1610 and 4170 kbp and for those causing cystitis they were between 1630 and 3740 kbp. These results could be compared with the previous ones conducted on different organisms.[34][35] Determining the genome sizes of bacterial genomes as revealed by endonuclease restriction enzyme, could provide an alternative parameter to be employed in taxonomic studies.[34] Results obtained in our study indicated the high heterogeneity of *E. coli* strains. Genome size estimation can also help gain more knowledge about the genome structure of UPEC isolated form children in the studied region and in doing so, we can adopt more effective strategies in the identification of native strains.

In the current work, 65 patterns for all the isolates (36 and 33 patterns for pyelonephritis and cystitis, respectively) based on drawn dendrogram were found (Figures 1, 2 and 3). The highest percentage of strains with 13 and 12 bands formed 25.2% and 23.3% of the samples respectively, and the least number of strains with the 19 and 8 bands formed 1.1% and the pattern No. 2 with 12 bands was repeated more than other pattern in this study. The similarities of the bands and patterns among different isolates can help detecting the location of dominant common genes of the pathogenic strains. Patterns obtained in this study revealed extensive genetic heterogeneity of *E. coli* in the region. The best description for the diversities is that the isolates representing the outbreak strains are not the recent progeny of a single (or common) precursor and do not have the same genotypes. The patterns obtained in the present study were comparable with those in previous reported ones.[28][36][37][38]

As demonstrated in some previous studies, host and organism related factors played an important role in the various clinical syndromes of urinary infections.[3][7][8] Most strains of UPEC expressed several pathogenic factors simultaneously. Some of the main factors are common in clinical syndromes, but their distributions vary, for example the various distributions of *P. fimberia* caused urinary tract infections.[39][40] Toxins and capsules are mainly associated with pyelonephritis,[39][41] but Adhesin particularly in children were more or less associated with cystitis.[1] Aerobactin system can be more seen among the strains causing pyelonephritis. This is probably related to the ability of *E. coli* strains to create cystitis, more than aggressive urinary tract infections because UPEC need fewer pathogenic factors to create cystitis. Generally, UPEC strains causing cystitis are different from those causing pyelonephritis,[1][42] and the differences of virulence factors among the strains cause diverse genetic patterns.

On the other hand, identical strains with the same virulence factors may have different geographical distributions which in turn cause different patterns (bands variation in number and size) of strains according to the clinical symptoms of urinary tract infections. Other reason that we could note for PFGE profiles alteration are mutations (insertion and deletion of DNA), gene rearrangement, or loss of genetic information. Point mutations occur most frequently in a genome and often go unrecognized, unless they occur in a gene coding area that alter the phenotypic behavior of the isolates, or in a restriction site that can be visualized through restriction techniques. Insertion can be seen as a large band that can not move as far along through the gel. On the contrary, because of the small strands of DNA in deletions, the band will transfer through faster than in insertion state.[43] In addition, the acquisition of new traits by horizontal gene transfer (HGT) is another driving force in the emergence of new bacterial variants. A current concern involving HGT that greatly impacts the human population is the transfer of antibiotic resistance genes between organisms which could produce different antibiogram patterns. The study of horizontal gene transfer in bacteria is extremely important because it provides insight into how bacteria exchange their characterization, which is of great consideration in designing treatments.[44]

Technical difficulties of laboratory methods can be assumed as another reason for such observed variations. It also depends on other factors such as individual person, laboratory setting up and equipments, reagents, interruptions or distractions and unknown reasons. In the present study specific pattern was not seen for each clinical syndrome, but some strains causing cystitis and pyelonephritis had identical genetic patterns (e.g. c1 and p28) which indicated that they arised from a clone. This may be due to common pathogenic factors in these two syndromes and in otherwise cases different clinical syndrome can be observed. Our results are comparable to some other reports. Rasko and colleagues showed that the isolates causing pyelonephritis possess special genes that only cause kidney infections, implying that strains causing cystitis and pyelonephritis are genetically different from each other and the genetic differences among factors cause different clinical syndromes. In contrast, Arthur et al. demonstrated that an acute pyelonephritis isolate could cause cystitis on reinfection. In another study, Guyer et al. found no significant differences between the groups uropathogenic isolates.[4]

The other reason why the strains with identical genetic patterns may cause different syndromes could be the progression of UTI from bladder to kidney indicating that the pyelonephritic organisms must make at least a transient passage through the bladder to reach the kidney. In fact, pili genes coding expression could have a crucial role so that if these genes are expressed, the bacteria have rising movement and cause pyelonephritis and in otherwise cases they cause bladder infections. Therefore, the strains with identical genetic patterns could cause different diseases. In the current work, we have also identified cystitis and pyelonephritis-specific bands although we can not rule out the possibility that there are functional homologues that are not possible to be identified by the PFGE techniques.

To the best of our knowledge, this is the first report of applying PFGE genotyping method to study the molecular epidemiology of UPEC infection and the genetic relationship between pyelonephritis and cystitis strains in Iran. In the present study, High genetic diversity of *E. coli* prevents the recognition of a special pattern in the creation of cystitis and pyelonephritis strains or any relationship between the corresponding strains, but in some cases the strains causing cystitis or pyelonephritis had identical genetic patterns which indicates that they arise from a clone and the different diseases could be the result of different gene factors.

It should be noted that although PFGE is taken as the gold standard technique in epidemiological studies, using this method alone does not fully confirm the similarity of two isolates. Therefore, the use of a second strains typing method or supplementary epidemiological analysis is helpful to understand the dominant gene and more analysis of the strains and how the genetic differences cause different diseases.
